# Clustering Pattern and Functional Effect of SNPs in Human miRNA Seed Regions

**DOI:** 10.1155/2018/2456076

**Published:** 2018-03-06

**Authors:** Sha He, Haiyan Ou, Cunyou Zhao, Jian Zhang

**Affiliations:** ^1^Bioinformatics Section, School of Basic Medical Sciences, Southern Medical University, Guangzhou, Guangdong 510515, China; ^2^Department of Medical Genetics, School of Basic Medical Sciences, Southern Medical University, Guangzhou, Guangdong 510515, China; ^3^Guangdong Key Laboratory of Biochip Technology, Southern Medical University, Guangzhou, Guangdong 510515, China; ^4^Guangdong Province Key Laboratory of Psychiatric Disorders, Southern Medical University, Guangzhou, Guangdong 510515, China

## Abstract

miRNAs are a class of noncoding RNAs important in posttranscriptional repressors and involved in the regulation of almost every biological process by base paring with target genes through sequence in their seed regions. Genetic variations in the seed regions have vital effects on gene expression, phenotypic variation, and disease susceptibility in humans. The distribution pattern of genetic variation in miRNA seed regions might be related to miRNA function and is worth paying more attention to. We here employed computational analyses to explore the clustering pattern and functional effect of SNPs in human miRNA seed regions. A total of 1879 SNPs were mapped to 1226 human miRNA seed regions. We found that miRNAs with SNPs in their seed region are significantly enriched in miRNA clusters. We also found that SNPs in clustered miRNA seed regions have a lower functional effect than have SNPs in nonclustered miRNA seed regions. Additionally, we found that clustered miRNAs with SNPs in seed regions are involved in more pathways. Overall, our results demonstrate that SNPs in clustered miRNA seed regions can take part in more intricate and complex gene-regulating networks with lower functional cost by functional complementarity. Moreover, our results also broaden current knowledge on the genetic variation in human miRNA seed regions.

## 1. Introduction

miRNAs are small noncoding RNAs of 20–22 nucleotides (nt) in length in their mature form, processed from a longer hairpin structure, that act as posttranscriptional gene regulators by either mRNA degradation or translational repression [1]. It is estimated that miRNAs regulate more than 30~60% of all protein-coding genes, thus building complex regulatory networks that participate in the control of most biological processes and are related to shaping phenotypic variability and disease development [2, 3]. miRNA-mediated gene regulation depends on perfect matching between the seven nucleotides of its seed region (nucleotides 2 through 8 from the 5′ end of the mature miRNAs) and the target sequence usually located at the 3′ untranslated regions (UTRs) of the regulated genes [4, 5]. With the rapid advance of genome sequencing technologies in recent years, many genetic variants have been identified in miRNA seed regions [6–9]. These genetic variants may disrupt the interactions between miRNAs and their targets or create new targets, rewiring the miRNA regulatory networks and causing diseases. Moreover, previous studies have proven that genetic variations in the seed regions have profound and broad effects on gene expression, phenotypic variation, and disease susceptibility in humans [10–13]. However, there is still little information on the distribution patterns and allele-dependent functional effect of the genetic variations in human miRNA seed regions.

Wang et al. [14] reported that the clustered miRNAs tend to be evolutionarily conserved and miRNAs in the same clusters tend to be coexpressed and regulate overlapping sets of target genes. Therefore, we hypothesized that clustered miRNAs tend to be more tolerant of genetic variations in the seed region due to functional complementary than are those nonclustered miRNAs. In order to test this hypothesis, we investigated the clustering patterns and functional effect of SNPs in human miRNA seed regions in this study. Our results demonstrate that SNPs in clustered miRNA seed regions can take part in more intricate gene-regulating networks with lower functional cost by functional complementarity.

## 2. Materials and Methods

### 2.1. Genome-Wide Identification of SNPs in Human miRNA Seed Regions

Genomic coordinates of human mature miRNAs were downloaded and extracted from miRBase version 21 (http://www.mirbase.org/). This release of miRBase comprises 1881 precursor sequences and 2813 mature miRNAs (2588 unique mature miRNA sequences). Genomic locations of miRNA seeds were determined from genomic locations of the 2nd and 8th bases of mature miRNAs. The SNP data for human assemblies GRCh38/hg38 were downloaded from NCBI dbSNP database (version 147, https://www.ncbi.nlm.nih.gov/SNP/). For each miRNA, we collected all SNPs in the seed regions from dbSNP using in-house Perl scripts.

### 2.2. Computational Predictions of Allele-Dependent miRNA Target and Percent Overlap

The miRNA sequences were downloaded from the miRBase (version 21). To determine the target gene spectrum for the reference and derived allele of the SNPs in the miRNA seed regions, we predicted targets on the human genome assembly (NCBI36/hg18, March 2006) using the online target prediction program, namely, TargetRank (http://hollywood.mit.edu/targetrank) [15]. The reference targets for a SNP were predicted using TargetRank to analyse the miRNA seed sequence carrying the reference allele of the SNP. On the other hand, the derived targets of a SNP were predicted when the derived allele of the SNP was in the miRNA seed sequence. We defined the common targets (overlapping genes) as predicted targets for both the reference and derived alleles of a SNP. Percent overlap between the reference and derived targets for a SNP in miRNA seeds was determined using cosine similarity [16], which is calculated by the total number of overlapping genes divided by the square root of the product of the number of targets of reference and derived alleles of a SNP. Taking the square root of the number of predicted targets reduces the influence of miRNAs with abnormally large numbers of targets and simultaneously normalizes the result, generating a score between 0 and 1.

### 2.3. Clustering Analysis of miRNAs with SNPs in Their Seed Region

The clustering information of human miRNAs was obtained from Wang et al. [14] to investigate the clustering patterns of the miRNAs with SNPs in their seed region. Specifically, clustering of miRNA genomic locations is determined if two neighboring miRNAs are located within 10 kb and are in the same strand. Based on this criterion, among all the 1881 precursor sequences and 2813 mature miRNAs annotated in humans, 352 miRNA genes including 634 mature miRNAs were grouped into 99 distinct clusters [14]. The significance of the difference in percentage of clustered miRNAs between miRNAs with or without SNPs in seed regions was calculated using the chi-square test. Additionally, a two-tailed Student's *t*-test was used for comparisons between the percent overlap of the clustered and nonclustered miRNAs with SNPs in seed regions. *P* value < 0.05 was considered statistically significantly.

### 2.4. Function and Pathway Analysis

The function annotation and pathway enrichment of the clustered and nonclustered miRNAs with SNPs in seed regions were performed using miRNA Enrichment Analysis and Annotation (miEAA) tool database (http://www.ccb.uni-saarland.de/mieaa_tool/) [17]. miEAA is a web-based system, which offers miRNA set enrichment analysis similar to gene set enrichment analysis (GSEA).

## 3. Results and Discussion

### 3.1. miRNAs with SNPs in Their Seed Region Are Significantly Enriched in Clusters

In total, we identified 1879 SNPs in 1226 (43.6%) human miRNA seed regions after mapping genetic variation onto human miRNA seed regions based on the genomic coordinates of SNPs in dbSNP human Build 147 and miRNAs in miRBase release 21 (Table S1). We found that most of the SNPs (1833 SNPs, 97.5%, Table S1) in miRNA seed regions were rare variants (defined as SNPs with minor allele frequency (MAF) < 5%). Recently, Torruella-Loran et al. [18] studied miRNA genetic variation in human populations and found that the seed regions tend to be depleted of high-frequency variants, which is consistent with our finding. We also found that there are 1587, 749, 340, 102, 31, and 4 miRNAs, which carry zero, one, two, three, four, and five SNPs, respectively, in their seed region (Figure S1, Table S1). This indicates that miRNA seed regions might be not so tolerant of genetic variants since most miRNAs have few or rare SNPs in their seed regions.

**Figure 1:**
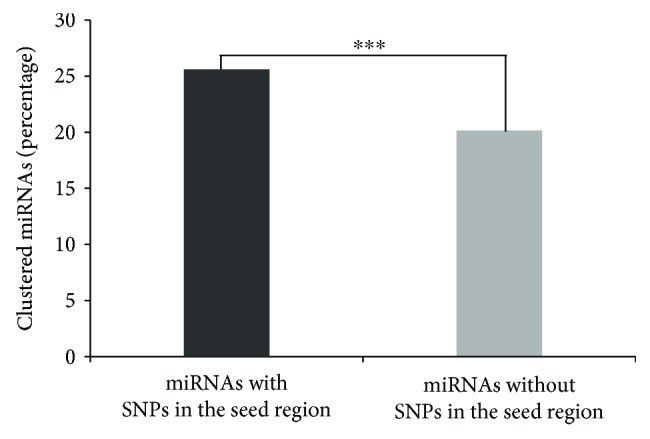
Clustering patterns of the miRNAs with SNPs in their seed region. Percentage of clustered miRNAs in miRNAs with or without variations in their seed region. miRNAs with SNPs in their seed region were significantly enriched in clusters. The *y*-axis is the percentage of miRNAs that are located in clusters in that group. ^∗∗∗^ *P* < 0.001.

Further, we investigated the clustering patterns of the miRNAs with SNPs in their seed regions. An interesting observation is that the miRNAs with SNPs in their seed region are significantly enriched in clusters (Figure 1, Table S2). For the 1226 human miRNAs with SNPs in their seed region, 314 (25.6%) of them are located in miRNA clusters, whereas among the 1587 human miRNAs without SNPs in their seed region, only 320 (20.2%) of them are located in miRNA clusters (*P* = 6.06 × 10^−4^, *χ* ^2^ test) (Table S2). miRNAs from the same cluster have the tendency to regulate the same sets of target genes and cooperatively repress expression levels of such genes [14]. Therefore, clustered miRNAs tend to be more tolerant of genetic variations in the seed region due to functional complementarity than are nonclustered miRNAs.

### 3.2. SNPs in Clustered miRNA Seed Regions Have a Lower Functional Effect

We speculated that the functional effect of genetic variation in clustered miRNA seed regions may be relatively low compared with that in nonclustered miRNA seed regions. The functional effect or “cost” of SNPs in miRNA seed regions involves the loss of regulatory control over previously targeted mRNAs and/or the acquisition of novel regulatory control over previously untargeted mRNAs [16].

In order to test this hypothesis, we calculated the percent overlap (cosine similarity) of predicted targets for the reference and derived allele of each SNP by TargetRank. A lower percent overlap indicates large overall differences between the targets of reference and derived allele. The average percent overlap between reference target sets and derived target sets for all the 1879 SNPs is only 15.8% (Table S1). By considering the importance of miRNAs in regulating gene expression, this result suggests that as few as one nucleotide substitution within the seed region of miRNAs will cause a significant functional effect.

**Figure 2:**
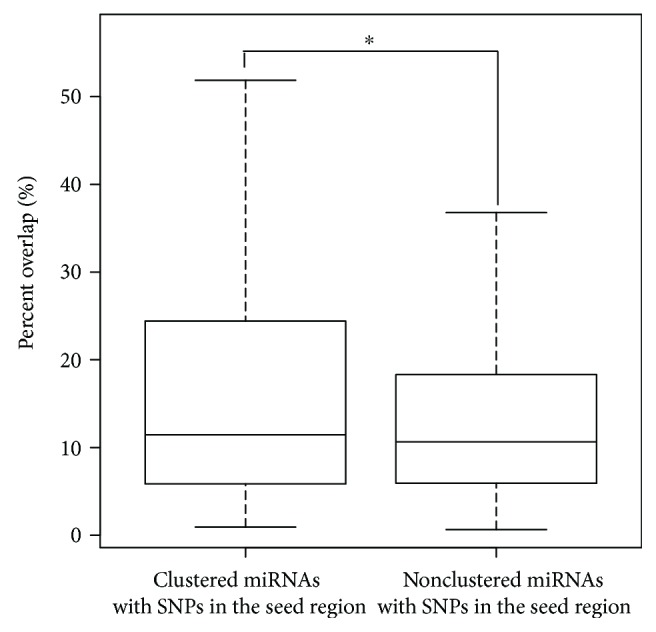
Difference in the functional effect of SNPs in clustered and nonclustered miRNA seed regions. ^∗^ *P* < 0.05.

Then, we compared the percent overlap of SNPs in clustered miRNA seed regions with that of SNPs in nonclustered miRNA seed regions. We found that the percent overlap of SNPs in clustered miRNA seed regions is much higher than that of SNPs in nonclustered miRNA seed regions (Student's *t*-test; *P* < 0.05) (Figure 2). Therefore, it indicates that SNPs in clustered miRNA seed regions will produce a lower functional effect, which may be a result of functional complementarity and relaxed selection or adaptive evolution.

### 3.3. Clustered miRNAs with SNPs in Seed Regions Are Involved in More Pathways

Additionally, we applied a gene set enrichment analysis to understand on which pathways the clustered and nonclustered miRNAs with SNPs in seed regions participate using miEAA [17].  Table 1 gives most enriched pathway information with *P* value < 0.01. Clustered miRNAs with SNPs in seed regions are involved in more pathways, such as Alzheimer disease amyloid secretase pathway, and TCR and IL signaling pathways, whereas nonclustered miRNAs with SNPs in seed regions are only enriched in TCR and Id signaling pathways (*P* < 0.01). From the point of evolution, a gene can allow more variations and obtain new function during evolution through gene duplication [19]. We guess that compared with nonclustered miRNAs, clustered miRNAs with SNPs in seed regions can take part in more intricate and complex regulating networks by obtaining SNPs in seed regions and functional complementarity of the members in the same cluster.

## 4. Conclusions

In this study, we took advantage of microRNA gene location and genetic variability obtained from miRBase 21 and dbSNP database to systematically identify all substitutions located in human miRNA seed regions and explore the clustering pattern and functional effect of these SNPs. In total, we have identified 1879 SNPs in 1226 human miRNA seed regions. We found that miRNAs with SNPs in their seed region are significantly enriched in clusters. We also found that functional cost of genetic variations in clustered miRNA seed regions was relatively low due to functional complementarity compared with that of variations in nonclustered miRNA seed regions. Additionally, we found that clustered miRNAs with SNPs in seed regions are involved in more pathways. Taken together, our results broaden current knowledge on the genetic variation in human miRNA seed regions and demonstrate that SNPs in clustered miRNA seed regions can take part in more intricate and complex networks with lower functional cost by functional complementarity.

## Figures and Tables

**Table 1:** Pathway enrichment analysis of clustered or nonclustered miRNAs with SNPs in seed regions by using miEAA.

Term	*P* value
Clustered miRNAs with SNPs in seed regions	
Alzheimer disease amyloid secretase pathway (P00003)	0.0010
Selenium (WP15)	0.0020
TCR signaling pathway (WP69)	0.0028
IL-3 signaling pathway (WP286)	0.0031
IL-1 signaling pathway (WP195)	0.0036
Endochondral ossification (WP474)	0.0047
IL-5 signaling pathway (WP127)	0.0056
Natural killer cell-mediated cytotoxicity (hsa04650)	0.0062
G protein signaling pathways (WP35)	0.0066
IL-6 signaling pathway (WP364)	0.0075
IL-2 signaling pathway (WP49)	0.0078
EPO receptor signaling (WP581)	0.0081
Ubiquitin proteasome pathway (P00060)	0.0098
Nonclustered miRNAs with SNPs in seed regions	
TCR signaling pathway (WP69)	0.0074
Id signaling pathway (WP53)	0.0097

## Supplementary Materials

Supplementary 1 Figure S1: SNPs in human miRNA seed regions. Number of miRNAs that carry zero, one, two, three, four, and five SNPs, respectively, in their seed region.

Supplementary 2 Table S1: genome-wide identification of SNPs in human miRNA seed regions.

Supplementary 3 Table S2: chi-square test of clustering patterns of miRNAs with SNPs in their seed region.
