# Supplementary figures and images for: Clustering Pattern and Functional Effect of SNPs in Human miRNA Seed Regions
Source: Int J Genomics. 2018 Mar 6;2018:2456076. doi: 10.1155/2018/2456076 (PMC5859846; doi:10.1155/2018/2456076)

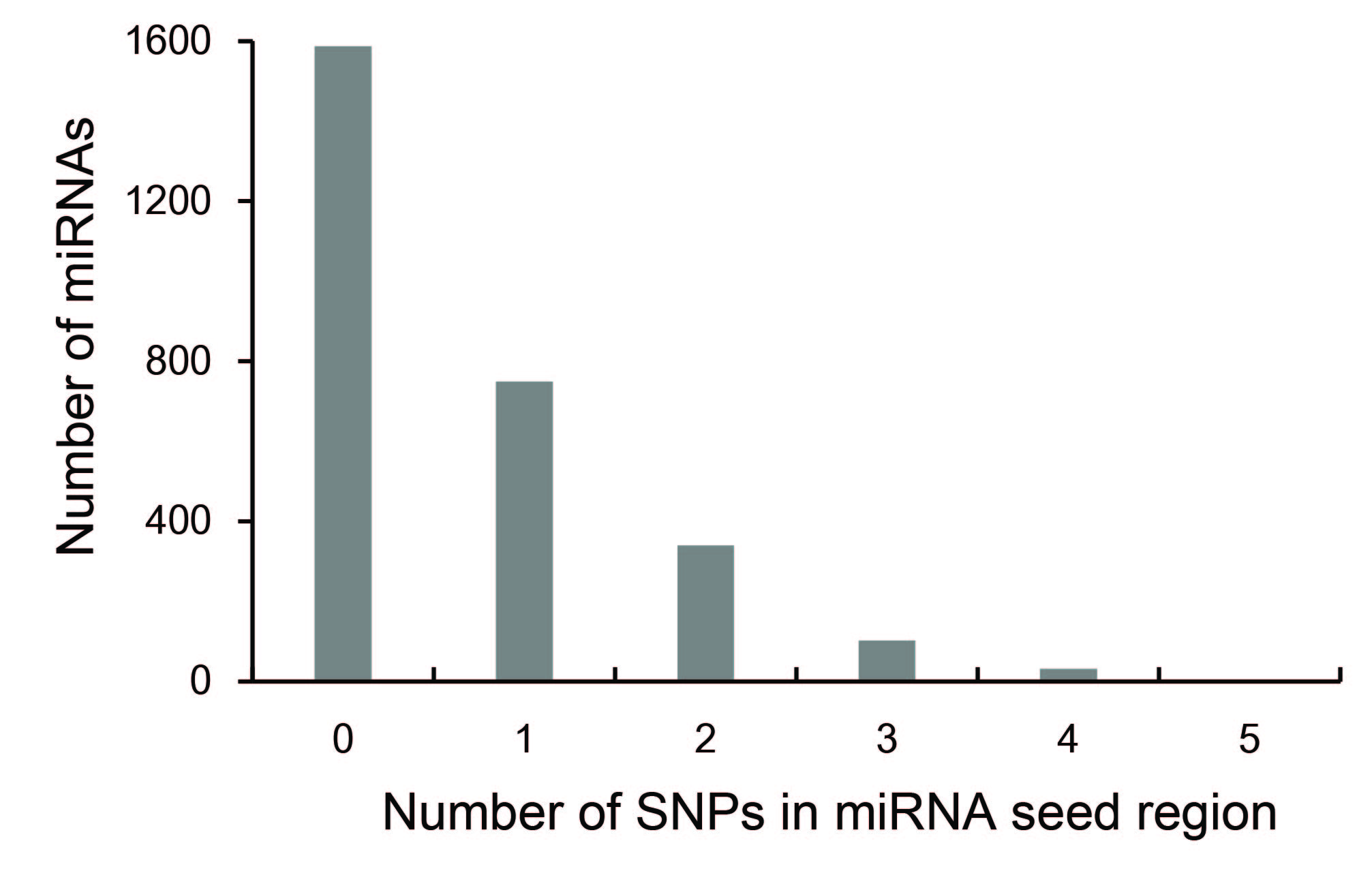

Supplement: Supplementary 1 — Figure S1: SNPs in human miRNA seed regions. Number of miRNAs that carry zero, one, two, three, four, and five SNPs, respectively, in their seed region. [file 2456076.f1.jpg]
