# Supplementary material for: Clustering Pattern and Functional Effect of SNPs in Human miRNA Seed Regions
Source: Int J Genomics. 2018 Mar 6;2018:2456076. doi: 10.1155/2018/2456076 (PMC5859846; doi:10.1155/2018/2456076)
Supplement: Supplementary 3 — Table S2: chi-square test of clustering patterns of miRNAs with SNPs in their seed region. [file 2456076.f3.docx]

Table 2. Chi-square test of clustering patterns of miRNAs with SNPs in its seed region.

|  | Clustered miRNA | Non-clustered miRNA | *P value* |
| --- | --- | --- | --- |
| Number of miRNAs with SNPs in seed region | 314 | 912 | 0.0006057 |
| Number of miRNAs without SNPs in seed region | 320 | 1267 |  |
